# XIST promotes apoptosis and the inflammatory response in CSE-stimulated cells via the miR-200c-3p/*EGR3* axis

**DOI:** 10.1186/s12890-021-01582-8

**Published:** 2021-07-09

**Authors:** Panfeng Chen, Ping Jiang, Jianing Chen, Yang Yang, Xiumei Guo

**Affiliations:** 1 Department of Respiratory and Critical Care Medicine, Tianjin First Central Hospital, No. 24 Fukang Road, Nankai District, Tianjin, 300192 China; 2 Department of Respiratory and Critical Care Medicine, Haihe Hospital, Tianjin, 300222 China; 3 Department of Orthopaedics, Baoding Second Central Hospital, Baoding, 072750 Hebei China

**Keywords:** XIST, miR-200c-3p, *EGR3*, Chronic obstructive pulmonary disease

## Abstract

**Background:**

Chronic obstructive pulmonary disease (COPD) is a disease that causes obstructed airways and abnormal inflammatory responses in the lungs. Early growth response 3 (*EGR3*) has been revealed to play a vital role in the regulation of the inflammatory response in certain diseases. We aimed to explore the role of *EGR3* and its upstream mechanism in COPD.

**Methods and result:**

In the present study, 16HBE cells were treated with cigarette smoke extract (CSE) to mimic the inflammatory response in vitro. RT-qPCR revealed that the expression of *EGR3* was upregulated in lungs from COPD patients. *EGR3* expression in 16HBE cells was increased by CSE treatment. Moreover, flow cytometry analysis and western blot analysis showed that *EGR3* downregulation inhibited 16HBE cell apoptosis. *EGR3* silencing decreased the protein levels of IL-6, TNF-α, IL-1β and COX2 in CSE-stimulated 16HBE cells. In addition, *EGR3* was targeted by microRNA-200c-3p (miR-200c-3p) in 16HBE cells. MiR-200c-3p expression was significantly decreased in lung tissues from COPD patients compared to that in healthy controls. Furthermore, miR-200c-3p bound to lncRNA X-inactive specific transcript (XIST) in 16HBE cells. Additionally, XIST expression was elevated in lung tissues from COPD patients. Rescue assays indicated that *EGR3* overexpression counteracted the effects of XIST downregulation on apoptosis and inflammation in CSE-stimulated 16HBE cells.

**Conclusion:**

The XIST/miR-200c-3p/*EGR3* axis facilitated apoptosis and inflammation in CSE-stimulated 16HBE cells. These findings may provide novel insight for treating COPD by alleviating lung inflammation.

**Supplementary Information:**

The online version contains supplementary material available at 10.1186/s12890-021-01582-8.

## Introduction

Chronic obstructive pulmonary disease (COPD) is a disease that causes obstructed airways and abnormal inflammatory responses in the lungs [1, 2]. COPD affects approximate 328 million people worldwide each year and causes 3.5–4 million deaths [3]. Smoking-induced chronic inflammation, tissue destruction and inhibition of tissue repair in the lungs are the major factors for COPD [4]. The inhalation of cigarette smoke activates pattern-recognition receptors, stimulating an innate immune response that activates the airway epithelial cells [5]. Activation of these cells triggers the release of various proinflammatory cytokines that recruit neutrophils perpetuate chronic inflammation in COPD [6, 7].

Early growth response 3 (*EGR3*), also known as PILOT, is a member of the EGR family of C2H2-type zinc-finger proteins [8]. Many studies have revealed that *EGR3* plays a critical role in the inflammatory response in various pathogeneses [9, 10]. The transcription factor *EGR3* is essential for the proliferation and the inflammatory response of B and T cells [10]. *EGR3* activates the transcription of proinflammatory cytokines, interleukin (IL)-6 and IL-8, to facilitate the inflammatory response in prostate cancer [11]. *EGR3* acts as an oncogene in lung cancer [12], and COPD has been indicated to worsen lung cancer prognosis [13]. Considering the role of *EGR3* in inflammation-associated diseases and in lung cancer as mentioned above, we hypothesized that *EGR3* exerts a significant effect in COPD.

Recently, the competing endogenous RNA (ceRNA) hypothesis has been widely proposed to play a pivotal role in COPD [14, 15]. The ceRNA hypothesis indicates that long noncoding RNA (lncRNA) transcripts containing miRNA-binding sites can stabilize mRNA expression by competing for shared microRNAs (miRNAs) [16]. MiRNAs are small noncoding RNAs that modulate gene expression by binding to the 3′ untranslated region (3′UTR) of target messenger RNAs (mRNAs) to degrade mRNAs [17, 18]. We hypothesized that *EGR3* is regulated by the lncRNA mediated ceRNA pattern in COPD.

In this study, the expression profile of *EGR3* in lung tissues of COPD patients and its function in inflammatory response of an in vitro COPD model were investigated. Moreover, the upstream ceRNA mechanism underlying *EGR3* mediated by lncRNA was explored. Our research may provide a potential novel direction for the clinical treatment of COPD.

## Materials and methods

### Patients and specimens

Lung specimens were collected from fifty-five patients with solitary non-small cell lung cancer receiving lobectomy or pneumonectomy combined with resection of a part of left atrium at Tianjin First Central Hospital between July 2016 and July 2018. The samples were divided into the following three groups: nonsmokers without COPD (n = 7; 5 men and 2 women; age range, 36–73 years; mean age, 55.1 ± 5.9 years old), smokers without COPD (n = 22; 16 men and 6 women; age range, 34–70 years; mean age, 52.6 ± 5.5 years old), and smokers with COPD (n = 26; 21 men and 5 women; age range, 31–74 years; mean age, 50.7 ± 5.3 years old). COPD was diagnosed through systemic physiological examinations according to the standard of the Global Initiative for Chronic Obstruct Lung Disease. Patients in the “smokers with COPD” group were first diagnosed with COPD and no other severe clinical disorders were observed. All nonsmokers without COPD received systemic physiological examinations and all parameters were within normal ranges. The smokers without COPD had a normal lung function at the time of admission and a history of smoking for 10.1–17.5 years. Patients were excluded if they suffered from asthma or other obstructive lung diseases. Written informed consents were obtained from all participants. The Ethics Committee of Tianjin First Central Hospital approved our study (approval number: 2020-011).

### Immunohistochemistry (IHC)

The airway tissues were inactivated with 3% hydrogen peroxide for 10 min at room temperature and blocked with 5% bovine serum albumin for 20 min followed by incubation with anti-EGR3 polyclonal antibody (1:200, ab232820, Abcam) at 4 °C overnight. Next, the sections were incubated with the horseradish peroxide-conjugated secondary antibody at room temperature for 2 h. Subsequently, EGR3 protein was visualized using diaminobenzidine and counterstained with hematoxylin. Images of stained airway tissues were photographed with an inverted fluorescence microscope (Olympus, Japan) and analyzed with Image Pro Plus 6.0 software.

### Cell culture and treatment

Exposure to cigarette smoke extract (CSE) can cause an abnormal inflammatory response in the small airways and alveoli, thereby accelerating the apoptosis of bronchial epithelial cells [19]. CSE-treated bronchial epithelial cells are widely used as an in vitro model of COPD [20–22]. The normal human bronchial epithelial cell line 16HBE was purchased from American Type Culture Collection (ATCC; USA) and cultured in RPMI-1640 medium (Invitrogen, USA) supplemented with 10% fetal bovine serum (FBS, Invitrogen) at 37 °C with 5% CO_2_. For cell treatment, different concentrations (1%, 2%, 3% and 4%) of cigarette smoke extract (CSE) were prepared using 10 cigarettes (Suyan, Jiangsu Cigarettes Company, Jiangsu, China) as previously described [23]. In brief, the smoke from 10 cigarettes (Furong, Changde Cigarette Company, Hunan, China) was bubbled using 25-mL media. The suspension was titrated to pH 7.4, filter-sterilized, and regarded as 100% CES. The CSE sample was diluted with phosphate-buffered saline (PBS) to concentrations of 1%, 2%, 3% and 4%, and then stored at − 80 °C. The 16HBE cells were treated with CSE for 24 h. Cells in the control group were treated with the same dose of PBS.

### Cell transfection

Short hairpin RNA against XIST (sh-XIST) or *EGR3* (sh-*EGR3*) was utilized to knock down XIST or *EGR3*, respectively. sh-NC served as the negative control for sh-XIST or sh-*EGR3*. Coding region of *EGR3* was inserted into the pcDNA3.1 vector to overexpress *EGR3* and empty pcDNA3.1 vector (Vector) was used as the negative control. MiR-200c-3p mimics (miR-200c-3p) was used for the overexpression of miR-200c-3p and NC mimics was regarded as the negative control. All vectors were constructed by GenePharma (Shanghai, China). 16HBE cells (1 × 10^5^ cells/well) were seeded in 24-well plates with 500 μL of RPMI-1640 medium (Invitrogen) in each well. When the cells reached 40–60% confluence, the aforementioned vectors were transfected into cells at a final concentration of 50 nM using Lipofectamine® 2000 reagent (Invitrogen) at 37 °C and 5% CO_2_ for 48 h under the manufacturer’s instructions. Additionally, the related sequences of oligonucleotide used in this research were shown in Additional file 1: Table S1.

### Reverse transcription-quantitative polymerase chain reaction (RT-qPCR) analysis

The airway tissues were dissected, and relative RNA expression was detected in airway tissues. Total RNA was extracted from airway tissues and 16HBE cells with TRIzol reagent (Takara, Japan). Reverse transcription for *EGR3* and XIST was performed with the High-Capacity cDNA Reverse Transcription Kit (Applied Biosystems, USA). Real-time PCR was performed using SYBR Green Real-Time PCR MasterMix (Toyobo, Japan) with glyceraldehyde-3-phosphate dehydrogenase (GAPDH) as the internal control. For the quantification of miR-200c-3p, total miRNA was extracted from tissues and cells using the miRNeasy RNA Isolation Kit (Qiagen, USA). Total extract was reverse transcribed into cDNA using the miScript RT Kit (Qiagen) with RNU6 (U6) as the internal control. Real-time PCR was performed with the Bio-Rad CFX96 qPCR system (Hercules, USA), and expression was calculated with the 2^−△△CT^ method. The related sequences of primers were shown in Additional file 1: Table S1.

### Enzyme-linked immunosorbent assay (ELISA)

Concentrations of IL-6, TNF-α, COX2 and IL-1βin culture supernatant collected from 16HBE cells under different treatments were determined using commercial ELISA kits (R&D Systems, USA).

### Bioinformatics analysis

MiRNAs that potentially target *EGR3* and lncRNAs that potentially bind to miR-200c-3p were predicted from starBase 2.0 website (http://starbase.sysu.edu.cn/). MiR-200c-3p and miR-429 were predicted to target *EGR3* according to the overlapping prediction results of RNA22, PicTar and TargetScan tools. LncRNAs (XIST, RRN3P2, AC120036.4) which potentially bind with miR-200c-3p were predicted under the conditions of medium stringency of CLIP data and low stringency of degradome data. Putative binding site between XIST and miR-200c-3p was obtained from starBase 2.0 website. Putative binding site between *EGR3* and miR-200c-3p was predicted by TargetScan 7.2 website (http://www.targetscan.org/vert_72/).

### RNA immunoprecipitation (RIP) assay

RNA immunoprecipitation (RIP) assays were performed with a Magna RNA Immunoprecipitation Kit (Millipore, MA, USA). 16HBE cells were lysed in RIPA buffer containing magnetic beads conjugated with anti-Ago2 or anti-IgG. Argonaute 2 (Ago2) is a catalytic component of the RNA-induced silencing complexes (RISCs) [24]. MiRNA precursors were transcribed, processed into mature miRNAs and loaded onto Ago2 protein to form the RISC. Thus, miRNAs and their bound RNAs can be identified by immunoprecipitation of Ago2. Then, the immunoprecipitated RNAs were isolated by TRIzol reagent, and the enrichment of *EGR3*, miR-200c-3p and XIST was analyzed by RT-qPCR.

### Luciferase reporter assay

The XIST sequence or the 3’UTR fragment of *EGR3* containing the predicted binding site for miR-200c-3p was subcloned into a pmirGLO-luciferase Target Expression Vector to construct the XIST wild-type (pmirGLO-XIST-Wt) vector or the *EGR3* 3’UTR wild-type (pmirGLO-*EGR3* 3’UTR-Wt) vector, respectively. XIST sequence or *EGR3* 3’UTR was directed-mutated by Genepharma and were inserted into the pmirGLO vector to construct the pmirGLO-*EGR3* 3’UTR-Mut or pmirGLO-XIST-Mut plasmids. 16HBE cells were cotransfected with pmirGLO-*EGR3* 3’UTR-Wt vector (or pmirGLO-XIST-Wt vector), pmirGLO-*EGR3* 3’UTR-Mut vector (or pmirGLO-XIST-Mut vector) and miR-200c-3p mimics (or NC mimics) using Lipofectamine 2000 for 48 h. A dual luciferase assay kit (Promega) was applied to investigate luciferase activity.

### Flow cytometry analysis

Flow cytometry analysis was applied to detect apoptosis of 16HBE cells. After being transfected with the indicated plasmids for 48 h, the cells were rinsed with PBS twice followed by resuspension in binding buffer. Next, the cells were stained with Annexin V-Fluorescein Isothiocyanate (FITC) and propidium iodide (PI) (BD Biosciences, USA) in a dark room for 20 min. Apoptosis was measured by flow cytometry using a BD FACSCalibur system (BD Biosciences). Q1 represents living cells; Q2 represents early apoptotic cells; Q3 represents late apoptotic cells; Q4 represents necrotic cells. Cell apoptosis rate = number of cells in Q2 + Q3/number of cells in all quadrants.

### Western blot analysis

Total proteins were extracted from 16HBE cells with radioimmunoprecipitation assay (RIPA) buffer (Beyotime, Jiangsu, China) containing protease inhibitors (Beyotime). Protein concentrations were determined with the bicinchoninic acid (BCA) Protein Assay Kit (Beyotime). Equal amounts of proteins were separated by 10% sodium dodecyl sulfate–polyacrylamide gel electrophoresis and then transferred onto nitrocellulose membranes (Millipore, USA). After blocking with 5% nonfat milk, the membranes were incubated with specific primary antibodies, including anti-*EGR3* (ab221711), anti-Bax (ab32503), anti-Bcl-2 (ab196495), anti-cleaved caspase-3 (ab32042), anti-IL-6 (ab233706), anti-TNF-α (ab183218), anti-COX-2 (ab179800), anti-IL-1β (ab2105) and anti-GAPDH (ab181602). After washing with PBS, the membranes were incubated with goat anti-mouse IgG (ab150077) and analyzed using an Odyssey infrared scanner (Li-Cor, USA). All antibodies were purchased from Abcam (Cambridge, USA).

### Statistical analysis

Data were processed using SPSS 17.0 statistical software and are shown as the mean ± standard deviation. Kolmogorov–Smirnov tests were performed for checking data normality. All data were normally distributed. Student’s t-test was applied for statistical comparison between two groups, while differences among more than two groups were estimated using one-way analysis of variance followed by Tukey’s post hoc test. Pearson’s correlation analysis was used to analyze the correlations between the expression of *EGR3* and miR-200c-3p, miR-200c-3p and XIST, and *EGR3* and XIST in the lung tissues of smokers. *p* < 0.05 was considered statistically significant.

## Results

### *EGR3* was upregulated in COPD lungs

**Fig. 1:**
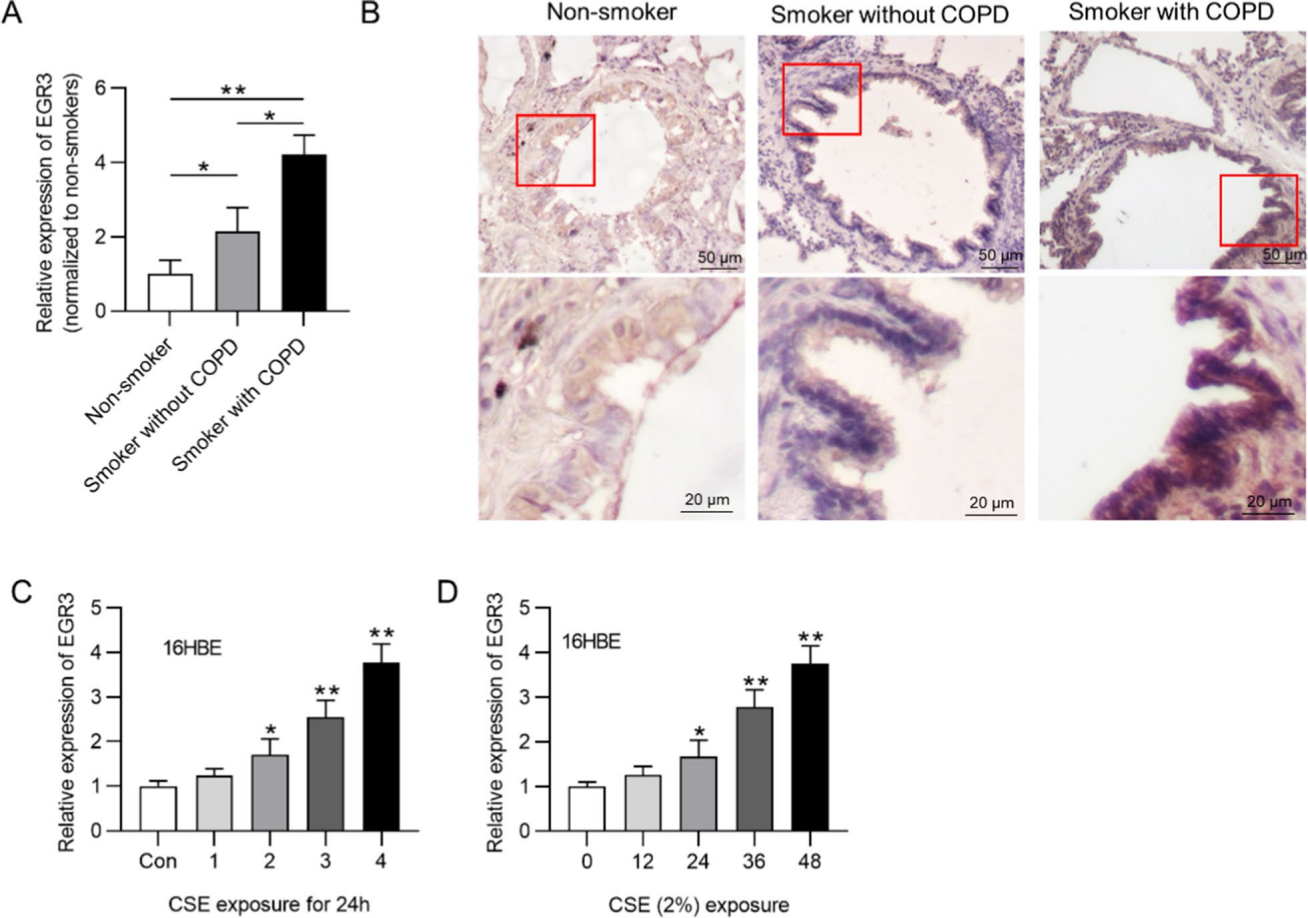
*EGR3* was upregulated in COPD lungs. **a** RT-qPCR was used to detect the expression of *EGR3* in lungs from nonsmokers (n = 7), smokers without COPD (n = 22) and smokers with COPD (n = 26). **b** IHC staining of EGR3 in airway tissues of nonsmokers, smokers without COPD, smokers with COPD. **c** The expression of *EGR3* in 16HBE cells treated with different concentrations of CSE (1, 2, 3, and 4%) for 24 h was evaluated by RT-qPCR (n = 3). **d** The expression of *EGR3* in 16HBE cells exposed to 2.5% CSE at 0, 12, 24, 36 and 48 h was measured by RT-qPCR (n = 3). *p < 0.05, **p < 0.01

First, we investigated the expression of *EGR3* in the clinical lung samples. The data revealed that *EGR3* was upregulated in the lung tissues of smokers compared with that in the lung tissues of nonsmokers. *EGR3* expression was higher in the lung tissues of smokers with COPD than smokers without COPD (Fig. 1a). Results of IHC staining assay of airway tissues revealed that EGR3 was mainly located in the cytoplasm of bronchial epithelial cells. EGR3 expression was significantly increased in the smoker group compared with nonsmoker group, and increased in COPD group compared with non-COPD group (Fig. 1b). Moreover, a normal human bronchial epithelial cell line (16HBE) was exposed to varying concentrations (1%, 2%, 3% and 4%) of CSE for 24 h. We found that *EGR3* expression was increased in CES-treated 16HBE cells in a dose-dependent manner (Fig. 1c). Additionally, *EGR3* expression was elevated in 16HBE cells exposed to 2% CSE in a time-dependent manner (Fig. 1d). All these experimental results suggested that the in vitro cellular model was successfully established, and 2% CSE treatment for 24 h was used for the following assays.

### *EGR3* downregulation inhibited apoptosis and the inflammatory response in CSE-treated cells

**Fig. 2:**
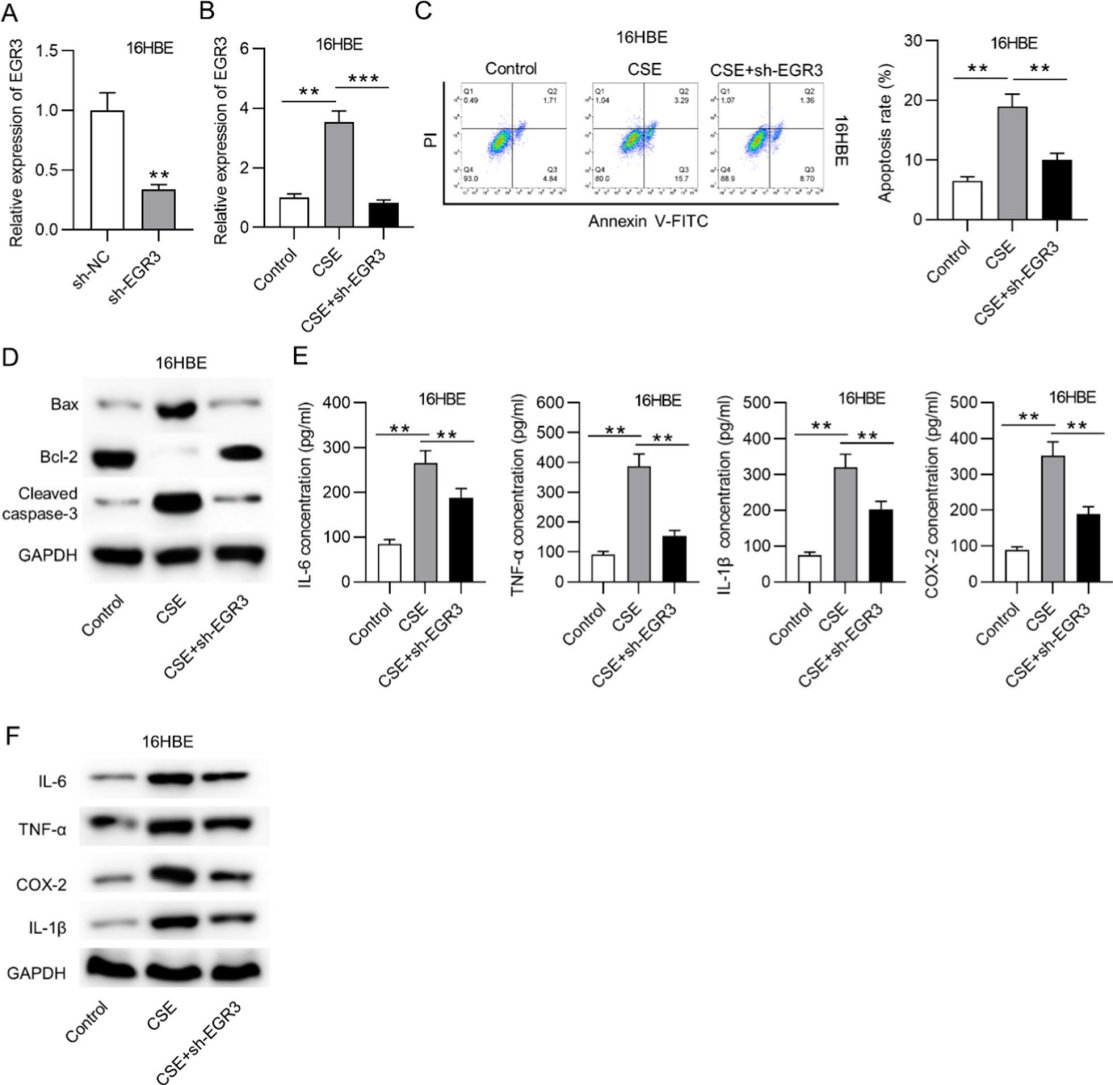
*EGR3* downregulation inhibited apoptosis and the inflammatory response in CSE-treated cells. **a** The transfection efficacy of sh-*EGR3* and sh-NC in 16HBE cells was tested by RT-qPCR (n = 3). **b** The expression of *EGR3* in CSE-treated 16HBE cells under indicated treatment was analyzed by RT-qPCR (n = 3). **c** Flow cytometry analysis was applied to assess apoptosis in CSE-treated cells (n = 3). **d** Western blot analysis was used to measure the levels of apoptosis-related proteins (Bax, Bcl-2 and cleaved caspase-3) in CSE-treated 16HBE cells (n = 3). **e** ELISA was used to measure the concentrations of proinflammatory factors (IL-6, TNF-α, IL-1β and COX2) in CSE-treated 16HBE cells (n = 3). **f** The protein levels of IL-6, TNF-α, IL-1β and COX2 in CSE-treated 16HBE cells were measured by western blot analysis (n = 3). **p < 0.01, ***p < 0.001

We then conducted loss-of-function assays to explore the function of *EGR3* in CSE-treated cells. *EGR3* expression was downregulated by transfection with sh-*EGR3* in basal 16HBE cells and CSE-stimulated 16HBE cells (Fig. 2a, b). Moreover, flow cytometry analysis showed that CSE treatment increased the apoptosis rate of 16HBE cells, and *EGR3* downregulation decreased the apoptosis rate of CSE-treated 16HBE cells (Fig. 2c). Additionally, the levels of apoptosis-related proteins were measured. The results revealed that Bax and cleaved caspase-3 protein levels were increased, and the Bcl-2 protein level was decreased by CSE treatment in 16HBE cells, and *EGR3* downregulation counteracted these effects (Fig. 2d). Moreover, the concentrations and protein levels of proinflammatory factors (IL-6, TNF-α, IL-1β and COX2) in 16HBE cells were increased by CSE treatment and repressed by knockdown of *EGR3* in the supernatants of CSE-treated cells (Fig. 2e, f).

### *EGR3* was a downstream target of miR-200c-3p

**Fig. 3:**
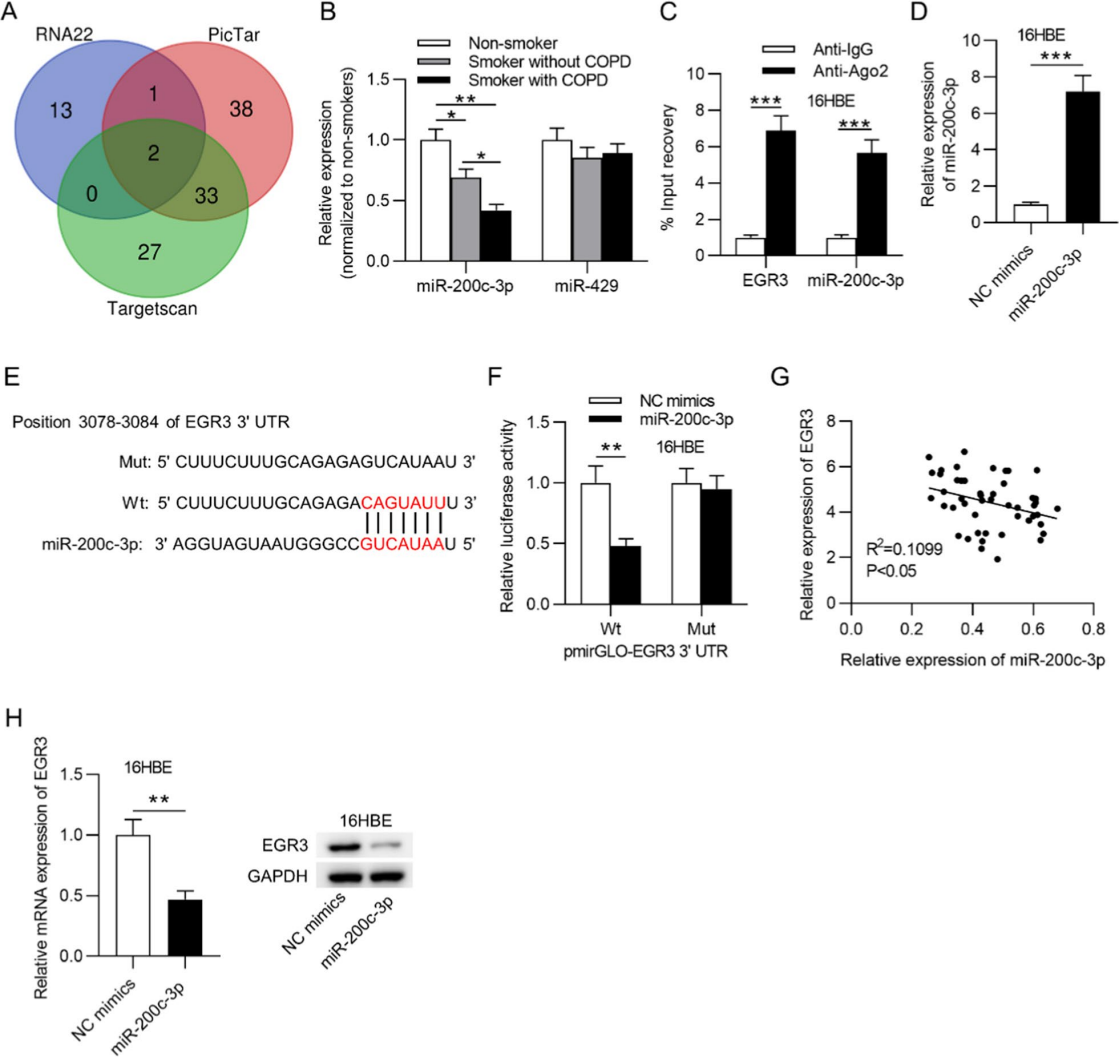
*EGR3* was a downstream target of miR-200c-3p. **a** Venn diagram showing the potential miRNAs possessing binding sites on the *EGR3* 3’UTR. **b** RT-qPCR was used to examine the expression of miR-200c-3p and miR-429 in lungs from nonsmokers (n = 7), smokers without COPD (n = 22), and smokers with COPD (n = 26). **c** RIP assay was conducted to reveal the relative enrichment of miR-200c-3p and *EGR3* precipitated by Ago2 normalized to that by IgG in 16HBE cells (n = 3). **d** RT-qPCR was used to measure miR-200c-3p expression in 16HBE cells transfected with NC mimics or miR-200c-3p mimics (n = 3). **e** The starBase database predicted the potential binding site of miR-200c-3p on the *EGR3* 3’UTR. **f** Luciferase reporter assay was used to assess the binding ability of miR-200c-3p and *EGR3* in 16HBE cells (n = 3). **g** The correlation between *EGR3* and miR-200c-3p expression in lung tissues of smokers (n = 48) was analyzed by Pearson’s correlation analysis. **h** The mRNA and protein levels of miR-200c-3p in response to miR-200c-3p overexpression in 16HBE cells (n = 3). *p < 0.05, **p < 0.01, ***p < 0.001

As the role of *EGR3* in COPD was corroborated above, the upstream mechanism underlying *EGR3* was explored. According to the starBase database (http://starbase.sysu.edu.cn/), miR-429 and miR-200c-3p were predicted to possess potential binding sites on the *EGR3* 3’UTR (Fig. 3a). Thereafter, RT-qPCR showed that miR-200c-3p expression was lower in the lung tissue of smokers compared to that of nonsmokers. Moreover, miR-200c-3p expression was lower in smokers with COPD than in smokers without COPD (Fig. 3b). In addition, a RIP assay was used to assess whether miR-200c-3p can bind with *EGR3* in 16HBE cells. From the results, we observed that *EGR3* and miR-200c-3p were both enriched in the Ago2 group but not in the IgG group (Fig. 3c), implying that miR-200c-3p targeted *EGR3* in 16HBE cells. Next, miR-200c-3p was overexpressed by transfection with miR-200c-3p mimics into 16HBE cells (Fig. 3d). The results of the luciferase reporter assay revealed that the overexpression of miR-200c-3p decreased the luciferase activity of *EGR3*-Wt but not that of *EGR3*-Mut in 16HBE cells (Fig. 3e, f). Moreover, *EGR3* expression was negatively correlated with miR-200c-3p expression in the lung tissues of 48 smokers (Fig. 3g). Furthermore, RT-qPCR and western blot analysis indicated that miR-200c-3p negatively regulated *EGR3* mRNA and protein levels in 16HBE cells (Fig. 3h).

### XIST bound with miR-200c-3p to regulate *EGR3*

**Fig. 4:**
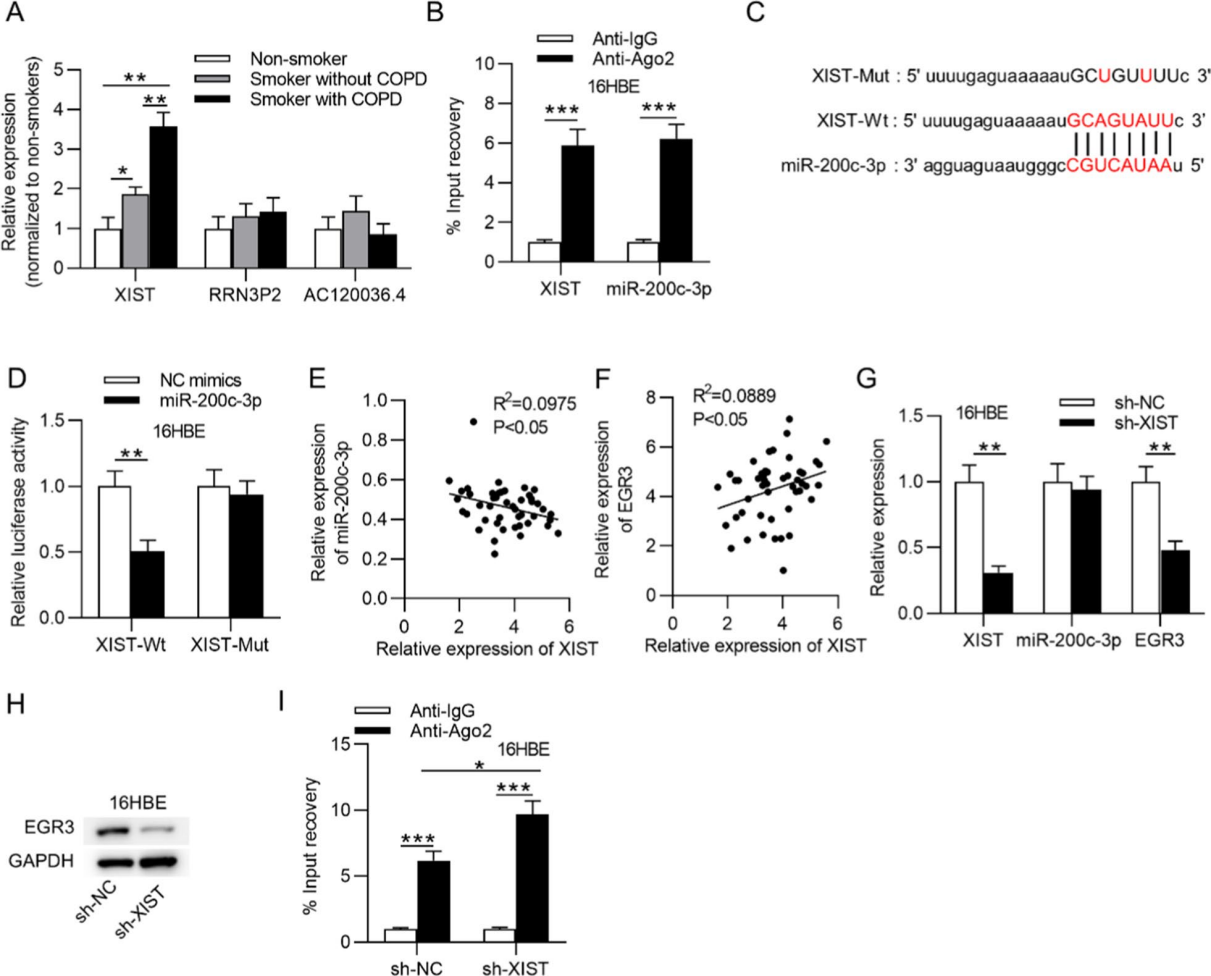
MiR-200c-3p interacted with XIST. **a** The levels of XIST, RRN3P2 and AC120036.4 in lung tissues from nonsmokers (n = 7), smokers without COPD (n = 22) and smokers with COPD (n = 26). **b** RIP assay was used to assess the relative enrichment of XIST and miR-200c-3p precipitated by Ago2 normalized to that by IgG in 16HBE cells (n = 3). **c** The starBase database showed the potential binding site for miR-200c-3p and XIST. **d** The binding ability between miR-200c-3p and XIST in 16HBE cells was verified by luciferase reporter assay (n = 3). **e** and **f**, Pearson’s correlation analysis demonstrated the correlation between XIST and *EGR3* (or miR-200c-3p) expression in lung tissues of smokers (n = 48). **g** The transfection efficacy of sh-XIST and sh-NC as well as the levels of miR-200c-3p and *EGR3* in 16HBE cells under XIST knockdown (n = 3). **h** The effect of XIST on *EGR3* protein levels in 16HBE cells (n = 3). **i** RIP assay was conducted to reveal the relative enrichment of *EGR3* precipitated by Ago2 normalized to that by IgG in 16HBE cells with silencing of XIST. *p < 0.05, **p < 0.01, ***p < 0.001

We wondered whether *EGR3* and miR-200c-3p are involved in the ceRNA network in 16HBE cells. The starBase database was used, and three lncRNAs (XIST, RRN3P2 and AC120036.4) were found to bind with miR-200c-3p. As shown in Fig. 4a, only XIST expression was significantly higher in the lung tissues of smokers than non-smokers, whereas the other two lncRNAs presented no differential expression difference in the lung tissues of smokers and nonsmokers. Furthermore, XIST expression was significantly higher in the lung tissues of smokers with COPD than smokers without COPD. The interaction between miR-200c-3p and XIST was then verified by RIP assay. Abundant enrichment of miR-200c-3p and XIST in 16HBE cells was observed in the Ago2 group but not in the IgG group (Fig. 4b). The binding sequence between XIST and miR-200c-3p was revealed in Fig. 4c. A luciferase reporter assay suggested that the luciferase activity of XIST-Wt was decreased in the miR-200c-3p mimic group compared to the NC mimic group, and the luciferase activity of XIST-Mut showed no evident change in either group (Fig. 4d). Pearson’s correlation analysis showed that miR-200c-3p expression was negatively associated with XIST expression and that *EGR3* expression was positively associated with XIST expression in the lung tissues of 48 smokers (Fig. 4e, f). Additionally, in response to XIST silencing in 16HBE cells, the levels of both XIST and *EGR3* were decreased, and the level of miR-200c-3p is not significantly affected (Fig. 4g). Furthermore, the protein level of *EGR3* was reduced by knockdown of XIST (Fig. 4h). After silencing of XIST, the enrichment of *EGR3* in the RISCs was increased, indicating the increase of *EGR3* binding to miR-200c-3p (Fig. 4I).

### *EGR3* overexpression reversed the XIST downregulation-mediated suppression of apoptosis and the inflammatory response in CSE-treated 16HBE cells

**Fig. 5:**
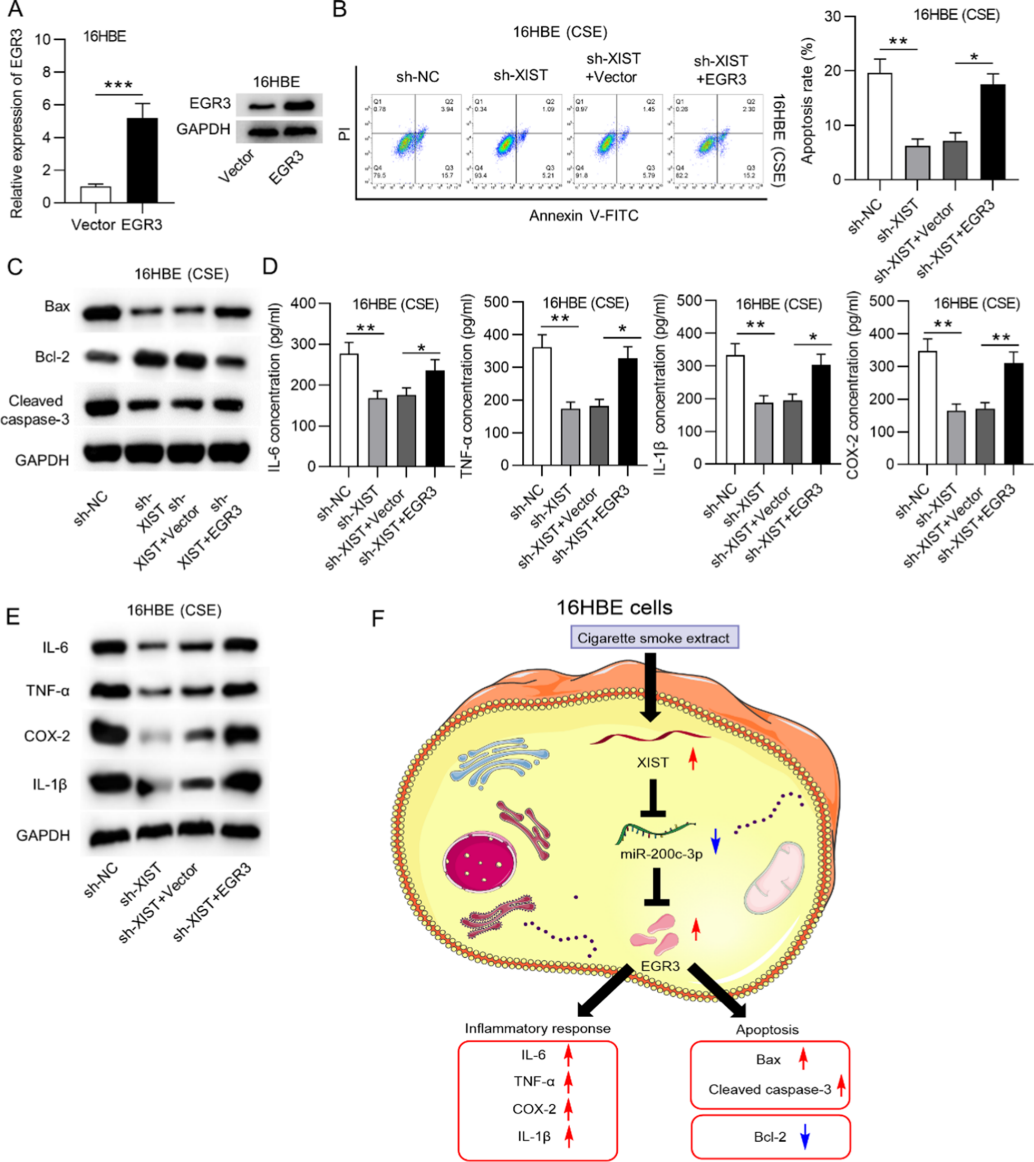
*EGR3* reversed the XIST downregulation-mediated suppression of apoptosis and inflammation in CSE-stimulated cells. **a** RT-qPCR and western blot analysis were used to evaluate the transfection efficacy of pcDNA3.1 or pcDNA3.1/*EGR3* in 16HBE cells (n = 3). **b** Flow cytometry analysis showed the apoptosis of CSE-treated 16HBE cells in each group (n = 3). **c** Western blot analysis was used to measure the protein levels of Bax, Bcl-2 and cleaved caspase-3 in CSE-treated 16HBE cells under the indicated transfection conditions (n = 3). **d** The concentrations of IL-6, TNF-α, IL-1β and COX2 in CSE-treated 16HBE cells under the indicated treatments were measured by ELISA (n = 3). **e** Western blot analysis showing the protein levels of IL-6, TNF-α, IL-1β and COX2 in CSE-treated 16HBE cells in each group (n = 3). **f** A schematic diagram: CSE induced XIST facilitates apoptosis and inflammatory response of 16HBE cells by binding with miR-200c-3p to upregulate *EGR3*. *p < 0.05, **p < 0.01, ***p < 0.001

Subsequently, we explored the role of XIST in CSE-treated 16HBE cells and investigated whether *EGR3* participates in XIST-mediated cellular behaviors. First, we transfected CSE-treated 16HBE cells with pcDNA3.1 or pcDNA3.1/*EGR3*. The results suggested that *EGR3* mRNA and protein levels were successfully overexpressed by transfection with pcDNA3.1/*EGR3* in 16HBE cells (Fig. 5a). Additionally, flow cytometry analysis showed that *EGR3* overexpression reversed the decrease of the cell apoptosis rate induced by XIST silencing in CSE-stimulated 16HBE cells (Fig. 5b). Moreover, the increase in Bcl-2 protein levels and the decrease in Bax and cleaved caspase-3 protein levels induced by XIST downregulation were rescued by *EGR3* overexpression in CSE-stimulated 16HBE cells (Fig. 5c). In addition, the decreased concentrations and protein levels of IL-6, TNF-α, IL-1β and COX2 induced by XIST knockdown were reversed by *EGR3* overexpression in CSE-treated 16HBE cells (Fig. 5d, e).

## Discussion

In our study, we found that CSE treatment promoted apoptosis and inflammation in 16HBE cells, which was consistent with clinical characterization of COPD. The results suggested the successful construction of an in vitro model of COPD. Previously, *EGR3* was indicated to exert vital roles in coronary heart disease and systemic lupus erythematosus [9, 25]. In the present study, *EGR3* was found to be upregulated in the lung tissues of smokers, especially in smokers with COPD. *EGR3* was mainly located in the chronical epithelial cells of airway tissues. *EGR3* was reported to promote antigen receptor signaling and control inflammation in adaptive immune responses and homeostasis [10]. In our research, *EGR3* silencing suppressed apoptosis and the inflammatory response of CSE-stimulated 16HBE cells. These findings suggested that *EGR3* promoted apoptosis and inflammation in CES-treated 16HBE cells.

Next, we aimed to explore the potential upstream mechanism of *EGR3* in 16HBE cells. Previously, multiple studies suggested that *EGR3* was regulated by several miRNAs at the post-transcriptional level [26–28]. In the current study, miR-200c-3p was predicted to be an upstream molecule of *EGR3*. MiR-200c-3p has been reported to be associated with the development of NSCLC [29]. MiR-200c-3p is a key regulator of bacterial lung infection-induced acute lung injury or acute respiratory distress syndrome [30]. We found that miR-200c-3p was downregulated in the lung tissues of COPD patients. Mechanistically, miR-200c-3p was validated to complementarily bind with the *EGR3* 3’UTR and negatively regulate *EGR3* mRNA and protein levels in 16HBE cells. In addition, miR-200c-3p expression was negatively correlated with *EGR3* expression in the lung tissues of smokers. The results suggested that *EGR3* was post-transcriptionally regulated by miR-200c-3p in 16HBE cells.

Moreover, miR-200c-3p was reported to interact with XIST in brain microvascular endothelial cells and breast cancer cells [31, 32]. Herein, after prediction by bioinformatics analysis, XIST was found to be a putative lncRNA that binds to miR-200c-3p. Mechanistic experiments revealed that XIST competitively bound with miR-200c-3p and thereby positively modulated *EGR3* mRNA and protein levels in 16HBE cells. Furthermore, the XIST expression was negatively correlated with the miR-200c-3p expression and positively correlated with the *EGR3* expression in the lung tissues of smokers. A whole transcriptome analysis of human lung tissue identifies XIST as a COPD-associated gene [33]. XIST is a positive regulator of inflammatory response, for example, XIST induces neuroinflammation by targeting the miR-544/STAT3 axis and the miR-137/TNFAIP1 axis in neuropathic pain [34, 35]. XIST mediates the inflammatory response of bovine mammary epithelial cells through the NF-κB/NLRP3 inflammasome pathway [36]. Moreover, XIST was widely reported to promote lung cancer progression [37–39]. In the present study, XIST expression was upregulated in the lung tissues of COPD patients. Rescue assays suggested that XIST downregulation inhibited apoptosis and the inflammatory response of CSE-treated 16HBE cells, and these effects were counteracted by *EGR3* overexpression. The results suggested that XIST regulated apoptosis and inflammation in CSE-treated 16HBE cells through modulating *EGR3* expression.

In conclusion, our study initially confirmed that the XIST and *EGR3* is upregulated and miR-200c-3p is downregulated in the airway tissues of smokers and COPD patients. We innovatively put forward that the XIST/miR-200c-3p/*EGR3* axis facilitates apoptosis and inflammation of CSE-treated 16HBE cells (Fig. 5f). Therefore, these data will provide a better understanding of XIST-mediated COPD pathogenesis and may provide potential novel insight for the treatment of COPD. However, our investigation on the lncRNA-mediated ceRNA regulatory network involved in COPD progression is still in preliminary phase with some limitations. First, we did not find the correlations between *EGR3* and parameters of lung function. Second, sample size limited the confidence of the present study to some degree. Third, the transformed bronchial epithelial cells we used in the present study might distinctly behave from primary bronchial epithelial cells isolated from COPD patients.

## Supplementary Information

**Additional file 1: Table S1.** The related sequences in this research.

## Data Availability

The datasets used and/or analyzed during the current study available from the corresponding author on reasonable request.
