# Supplementary material for: XIST promotes apoptosis and the inflammatory response in CSE-stimulated cells via the miR-200c-3p/EGR3 axis
Source: BMC Pulm Med. 2021 Jul 9;21:215. doi: 10.1186/s12890-021-01582-8 (PMC8268373; doi:10.1186/s12890-021-01582-8)
Supplement: Supplementary file 1 — Additional file 1: Table S1. The related sequences in this research. [file 12890_2021_1582_MOESM1_ESM.docx]

**Supplementary table 1. The related sequences in this research.**

| **Target** |  | **Sequence (5′-3′)** |
| --- | --- | --- |
| EGR3 primer | Forward: | GACATCGGTCTGACCAACGAG |
|  | Reverse: | GGCGAACTTTCCCAAGTAGGT |
| XIST primer | Forward: | AGACTACAGGATGAATTTGGAG |
|  | Reverse: | ACTCTTCACTCCTCTAAATCCA |
| RRN3P2 primer | Forward: | TGTAAAACGACGGCCAGT |
|  | Reverse: | CAGGAAACAGCTATGACC |
| AC120036.4 primer | Forward: | GGCTGAAAGCTGTTTGCCA |
|  | Reverse: | CAGAGGGATAGAGGGGCTGT |
| miR-200c-3p primer | Forward: | AATACTGCCGGGTAATGATGGA |
|  | Reverse: | CTCTACAGCTATATTGCCAGCCAC |
| miR-429 primer | Forward: | GGGGGTAATACTGTCTGGT |
|  | Reverse: | TGCGTGTCGTGGAGTC |
| sh-NC |  | CCGGCAACAAGATGAAGAGCACCAACTCGAGTTGGTGCTCTTCATCTTGTTGTTTTTG |
| sh-XIST |  | AUAACAGUAAGUCUGAUAGAGGACA |
| sh-EGR3 |  | CCGGCCCATTACAATCAGATGGCTACTCGAGTAGCCATCTGATTGTAATGGGTTTTTG |
| NC mimics |  | CAGUACUUUUGUGUAGUACAAA |
| miR-200c-3p mimics |  | UAAUACUGCCGGGUAAUGAUGGA |
